# Infection importation: a key challenge to malaria elimination on Bioko Island, Equatorial Guinea

**DOI:** 10.1186/s12936-015-0579-5

**Published:** 2015-02-05

**Authors:** John Bradley, Feliciano Monti, Andrea M Rehman, Christopher Schwabe, Daniel Vargas, Guillermo Garcia, Dianna Hergott, Matilde Riloha, Immo Kleinschmidt

**Affiliations:** MRC Tropical Epidemiology Group, London School of Hygiene and Tropical Medicine, London, UK; Medical Care Development International, Malabo, Equatorial Guinea; Medical Care Development International, Silver Spring, MD USA; Ministry of Health and Social Welfare, Malabo, Equatorial Guinea

**Keywords:** Malaria, Importation, Travel, Migration, Elimination

## Abstract

**Background:**

The impact of importation of falciparum malaria from mainland Equatorial Guinea on malaria infection in non-travellers and travellers on Bioko Island was examined.

**Methods:**

Malaria indicator surveys were conducted in 2013 and 2014 to assess the association between malaria infection and travel to the mainland. Infection in non-travellers was compared in neighbourhoods of high travel and neighbourhoods of low travel. Boat passengers leaving from and arriving on the island were tested for infection.

**Results:**

Children who had travelled to the mainland in the previous eight weeks were at greater risk of infection than those who had not travelled (56 *vs* 26% in 2013; 42 *vs* 18% in 2014). Children who had not travelled, living in localities with the highest proportion of travellers, were significantly more likely to be infected compared to those in localities with the smallest proportion of travellers (adjusted odds ratios 7.7 (95% CI 2.3-25) and 5.3 (95% CI 2.5-11) in 2013 and 2014, respectively). Infection in arriving boat passengers was substantially higher than in those departing (70 *vs* 38%, *p* = 0.017).

**Discussion:**

Malaria importation by travellers poses a serious public health challenge affecting non-travellers as well as travellers.

## Background

The importation of pathogens through population movement has long been seen as a risk factor in the transmission of infectious diseases, including malaria [1-4], especially for island populations [5-7]. This study, examined whether the importation of *Plasmodium falciparum* malaria parasites by travellers from the mainland part of Equatorial Guinea (EG) to Bioko, the main island of EG, contributed to the malaria burden in Bioko, in travellers and non-travellers. The Bioko Island Malaria Control Project (BIMCP) was launched in 2004 using a wide range of control measures with the aim of substantially reducing, and ultimately eliminating malaria from the island [8,9]. Substantial gains have been achieved in the past ten years, and the malaria burden is now considerably lower than on mainland EG [10]. Despite these gains malaria is far from being eliminated from the island [9,11,12].

**Figure 1:**
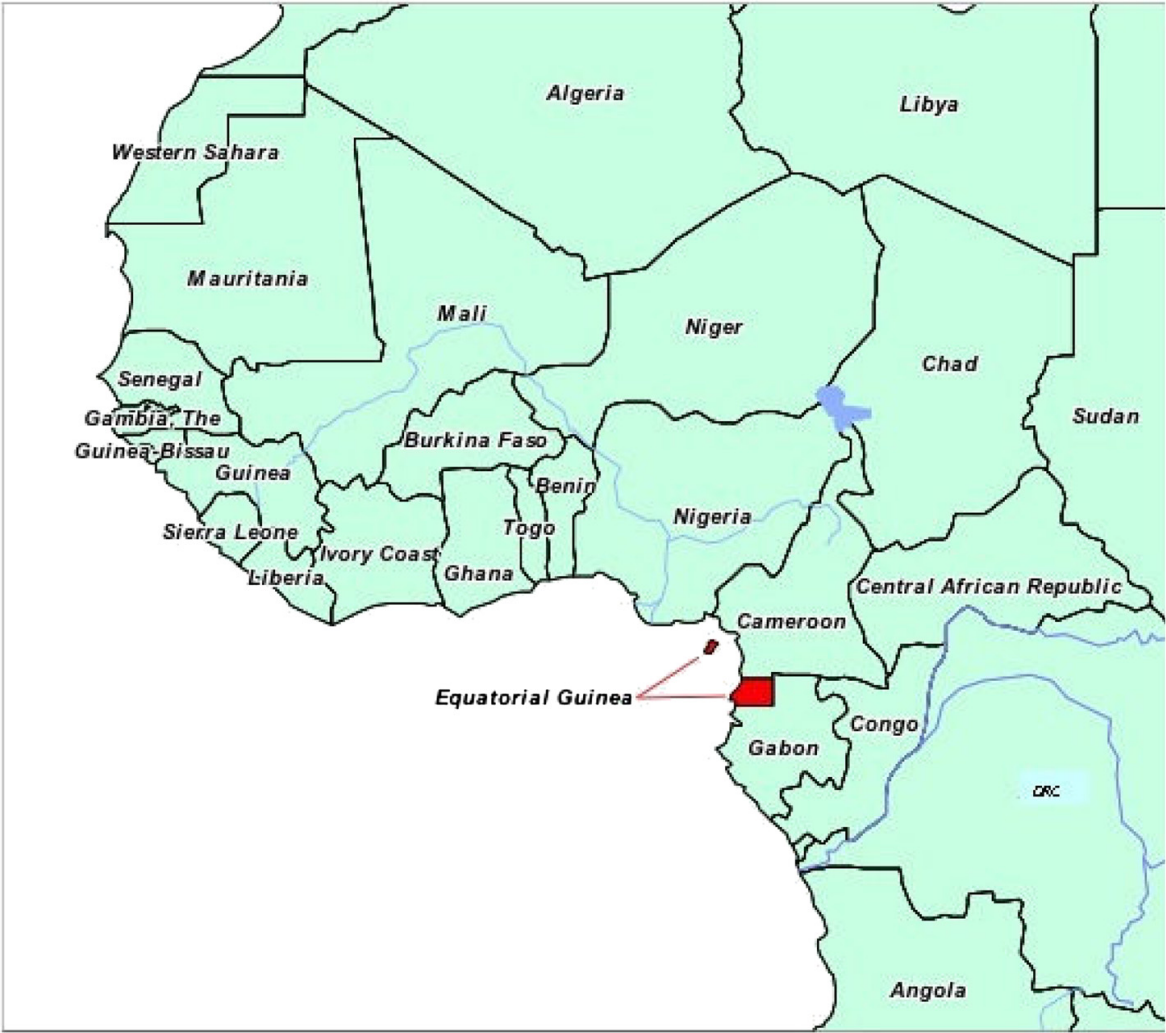
**Map of Bioko Island and mainland Equatorial Guinea.**

Bioko is located 32 km off the coast of Cameroon (Figure 1) with a population of approximately 250,000. Both the island and mainland EG have year-round malaria transmission. There are four boat sailings per week and approximately ten flights per day between Malabo on Bioko and Bata on mainland Equatorial Guinea, resulting in around 21,000 people arriving on Bioko every month from the mainland. This study is based on data from annual household malaria indicator surveys (MIS) in 2013 and 2014 and surveys of travellers on boats. Both the *vulnerability* (i.e., the rate of importation of infected individuals) and the *receptivity* (i.e., the potential for these travellers to transmit malaria to others) of Bioko to imported malaria infection are assessed in this study [13].

Bioko has received intensive malaria control as part of the BIMCP since 2004. As a result prevalence of *P. falciparum* malaria in two to 14 year old children fell from 45% before the start of interventions to 14, 28 and 18% in 2012, 2013 and 2014, respectively; moderate to severe anaemia (Hg <8 g/dL) in two to 14 year old children declined from 15 to 2% between 2004 and 2014 and all-cause under-five mortality reduced from 152 per 1,000 births to 55 per 1,000 in the first four years post intervention [8]. A similar package of interventions was introduced on the mainland in 2007, but funding restrictions considerably limited their scope [10].

## Methods

### Data collection

Cross-sectional household Malaria Indicator Surveys (MIS) have been conducted yearly on Bioko since the start of the BIMCP in 2004 [8,9,12,14-16]. Monitoring the impact of the BIMCP has been based on a system of 18 sentinel sites situated throughout the island. Houses were randomly sampled in each site from pre-compiled lists. Sample size was powered to show a change in prevalence of infection from 20 to 17% between years, assuming a design effect of 2.5. The survey instrument was adapted from the standard MIS developed by the Roll Back Malaria Monitoring and Evaluation Reference Group [17]. All members of a sampled household who were present were tested for *P. falciparum* using a rapid diagnostic test (RDT) (Carestart, AccessBio Inc, Monmouth, USA), subject to informed written consent from a caregiver. Participants testing RDT positive, with haemoglobin <8 g/dL or who were febrile were referred to a local clinic for appropriate treatment (anti-malarial, antipyretic or iron supplementation). In 2013 and 2014 respondents were asked if they had travelled to the mainland in the previous eight weeks.

A passenger survey was carried out on the boat *San Valentín* in December 2013 during one of its twice weekly sailings from Bata (EG mainland) to Malabo (Bioko) and from Malabo to Bata. Subject to informed written consent (from a caregiver in the case of children) passengers were given an RDT test and asked how many times they travelled between Bioko and the mainland in 2013 and how long they planned to stay at their destination. Passengers testing positive were offered treatment.

### Statistical analysis

For each site, for each of the two study years, the proportion of residents in the sentinel site who had travelled to the mainland in the previous eight weeks was calculated. The association between a survey respondent’s malaria infection status and whether they had travelled to the mainland was investigated. The relationship between infection in non-travellers and the proportion of residents in the sentinel site in which they lived who had travelled to the mainland was also examined. Analyses were done separately for children aged two to 14 and those aged 15 or over. Using logistic regression, adjusted odds ratios were calculated to control for the following confounders: site level seroconversion rate (SCR) measured in 2008 [18] as a measure of underlying transmission intensity, site level IRS coverage, age, use of a mosquito net, quintile of an asset based socio-economic score (SES) calculated by principal component analysis (PCA), and living in a house with closed eaves. Random effects models were used to account for correlation of responses within sentinel site. Linear regression was performed of site level prevalence of infection in non-travellers on site level proportion of residents who reported travelling to the mainland in the last eight weeks. A *z*-test of proportions was used to compare prevalence of infection in boat passengers travelling to Bioko from the mainland to the prevalence in those travelling in the opposite direction. All analyses were done using Stata software version 13 [19].

### Ethics and informed consent

Ethics approval for the surveys was granted by the Equatorial Guinea Ministry of Health and Social Welfare and the Ethics Committee of the London School of Hygiene and Tropical Medicine (approval number 5556). Informed written consent was given by each survey participant or, in the case of children, a responsible adult. In the case of participants being unable to read, the text was read and explained to them, and consent was confirmed by an independent witness identified on the consent form.

## Results

### Infection in travellers and non-travellers

The proportion of residents in a sentinel site who reported travelling to mainland EG in the previous eight weeks was 8.1% (range 0.9 to 16%) and 8.7% (range 0.5 to 16%) in 2013 and 2014, respectively. In 2013, travelling to the mainland was less common amongst children aged two to 14 years (6.2%) than it was amongst those aged 15 or over (9.6%). Travel was more common in those in the top quintile of SES (9.8%) than in those in the bottom quintile (3.9%) and more common in those who lived in urban sites (10.9%) than in those who lived in rural sites (4.0%). Similar results were obtained in 2014 (Table 1).

**Table 1:** **Proportion of respondents who travelled to mainland Equatorial Guinea in 2013 and 2014 by demographic group**

**Demographic group**	**% of survey respondents who travelled to the mainland in previous 8 weeks in 2013 (N)**	**% of survey respondents who travelled to the mainland in previous 8 weeks in 2014 (N)**
Overall	8.1% (20,536)	8.7% (20,880)
Children aged 2-14	6.2% (7,555)	6.1% (8,039)
Aged 15 or over	9.6% (11,498)	10.9% (11,436)
Living in urban sites	10.9% (12,080)	11.9% (12,309)
Living in rural sites	4.0% (8,456)	4.1% (8,571)
Highest SES quintile	9.8% (4,686)	14.3% (5,496)
Lowest SES quintile	3.9% (2,499)	4.6% (2,974)

Malaria infection prevalence on Bioko in 2013 was 27.8 and 18.2% in two to 14 year olds and over 15 s, respectively; the corresponding figures in 2014 were 18.6 and 12.2%. Malaria infection was strongly associated with travel to the mainland (Table 2). Children who had travelled to the mainland in the previous eight weeks were at greater risk of infection than those who had not travelled (56 *vs* 26%, adjusted OR 3.0 (95% CI 2.3-4.1) in 2013; 42 *vs* 18%, adjusted OR 3.8 (95% CI 2.9-4.9) in 2014). Amongst survey participants aged 15 or over, there was a similar association between malaria infection prevalence and recent travel to mainland EG: 28 *vs* 17%, adjusted OR 1.8, (95% CI 1.4-2.2) in 2013; 19 *vs* 12%, adjusted OR 1.8 (95% CI 1.4-2.2) in 2014.

**Table 2:** **Associations between malaria infection and travel to the mainland in 2013 and 2014**

**Year**	**Age group years**	**Travelled to mainland EG in previous 8 weeks**	**Prevalence of** ***P. falciparum*** **infection**	**Odds ratio (95% CI)**	***p*** **-value**	Adjusted odds ratio* (95% CI)	***p*** **-value**
			**% (N)**				
2013	2-14	No	26.4 (5,579)	1			
		Yes	56.4 (252)	2.80 (2.14-3.67)	<0.001	3.04 (2.25-4.09)	<0.001
	>15	No	17.2 (6,322)	1			
		Yes	27.8 (586)	1.65 (1.35-2.01)	<0.001	1.77 (1.42-2.20)	<0.001
2014	2-14	No	17.7 (6,810)	1		1	
		Yes	41.7 (288)	3.33 (2.59-4.29)	<0.001	3.77 (2.90-4.90)	<0.001
	>15	No	11.7 (6,618)	1		1	
		Yes	19.0 (626)	1.70 (1.37-2.10)	<0.001	1.77 (1.41-2.22)	<0.001

*Adjusted for IRS coverage, net use, age, site level SCR, SES and sleeping in a house with closed eaves.

### Effect on non-travellers living in neighbourhoods with a high proportion of travellers

**Figure 2:**
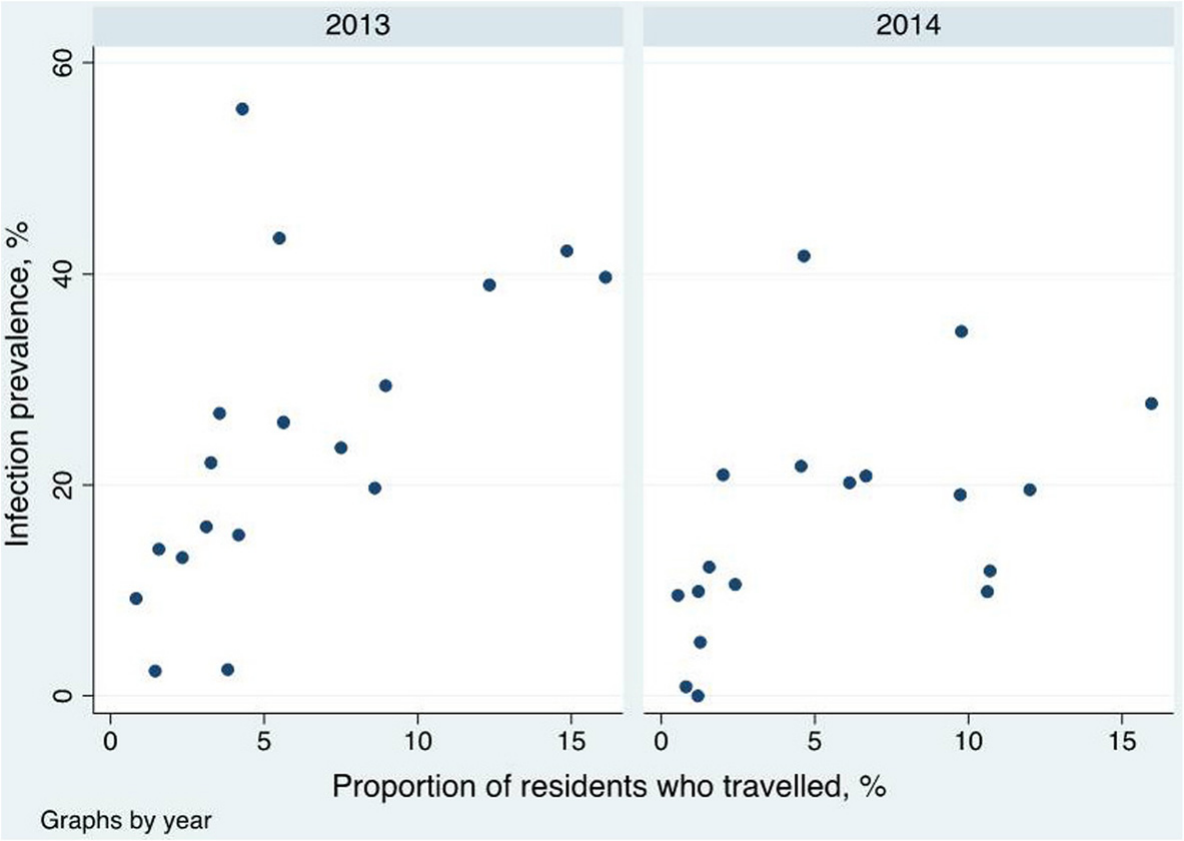
**Infection prevalence in non-travelling children versus the proportion of residents who travelled to Equatorial Guinea mainland.** Scatter plot of infection prevalence in non-travelling children 2 to 14 years old *versus* the proportion of residents who travelled to EG mainland in the 8 weeks preceding the survey, by sentinel site and by year, Bioko, EG 2013–2014. Linear regression coefficients for site level infection prevalence in non-travellers on proportion of residents who travelled are 2.0% per % travelled (95% CI, 0.61-3.4) for 2013 and 1.1% per % travelled (95% CI 0.03-2.2) for 2014.

In both the two to 14 year and the 15 and over age groups, infection with malaria in non-travellers was significantly associated with the proportion of people in their community who travelled to the mainland (Table 3 and Figure 2). Children who had not travelled in the previous eight weeks living in localities with the highest proportion of travellers were more likely to be infected with malaria parasites compared to those in localities with the smallest proportion of travellers (38 *vs* 10%, adjusted OR 7.7 (95% CI 2.3- 25) in 2013; 19 *vs* 5%, adjusted OR 5.3 (95% CI 2.5-11) in 2014). For those 15 and over who had not travelled, infection prevalence in those living in areas with the highest proportion of travellers compared to those in areas with the smallest proportion of travellers ranged from 23 to 8%, adjusted OR 3.1 (95% CI 1.4-7.0) in 2013, and 12 to 4%, adjusted OR 2.5 (95% CI 1.3-4.4) in 2014 (Table 3 and Figure 2).

**Table 3:** **Associations between infection in non-travellers and percentage of site residents who travelled to mainland**

**Year**	**Age group years**	**% of site residents who travelled to mainland EG in the previous 8 weeks**	**Prevalence of** ***P. falciparum*** **infection in non-travellers**	**Odds ratio (95% CI)**	***p*** **-value**	Adjusted odds ratio* (95% CI)	***p*** **-value**
			**% (N)**				
2013	2-14	<3.2% (5 sites)	10.2 (1,268)	1	0.001	1	0.004
		3.2% to <5% (5 sites)	28.9 (795)	2.58 (1.08-6.19)	ᅟ	2.50 (1.01-6.01)	ᅟ
		5 to <9% (4 sites)	26.5 (1,835)	3.60 (1.47-8.85)	ᅟ	5.07 (1.88-13.65)	ᅟ
		> = 9% (4 sites)	38.2 (2,261)	5.71 (2.33-14.01)	ᅟ	7.65 (2.34-24.96)	ᅟ
	>15	<1.5% (5 sites)	7.7 (1,188)	1	0.002	1	0.010
		1.5 to <5% (5 sites)	18.7 (992)	2.28 (1.14-4.60)	ᅟ	1.96 (1.04-3.67)	ᅟ
		5 to <10% (4 sites)	17.9 (2,280)	2.95 (1.26-4.54)	ᅟ	3.19 (1.60-6.35)	ᅟ
		> = 10% (4 sites)	22.7 (2,845)	3.84 (1.88-7.83)	ᅟ	3.10 (1.39-6.94)	ᅟ
2014	2-14	<1.5% (5 sites)	5.1 (942)	1	<0.001	1	<0.001
		1.5 to <5% (5 sites)	23.7 (946)	5.97 (2.56-13.88)		5.80 (3.18-10.59)	
		5 to <10% (4 sites)	22.0 (2,121)	7.14 (2.96-12.20)		3.98 (2.07-7.66)	
		> = 10% (4 sites)	18.9 (3,263)	4.60 (1.92-11.04)		5.31 (2.51-11.22)	
	>15	<1.5% (5 sites)	3.9 (875)	1	<0.001	1	<0.001
		1.5 to <5% (5 sites)	14.8 (911)	4.07 (2.35-7.05)		3.41 (2.02-5.76)	
		5 to <10% (4 sites)	14.6 (2,094)	4.06 (2.34-7.07)		2.39 (1.38-4.45)	
		> = 10% (4 sites)	12.3 (3,489)	3.07 (1.78-5.29)		2.45 (1.27-4.38)	

*Adjusted for IRS coverage, net use, age, site level SCR, SES, and sleeping in a house with closed eaves.

### Results from boat passenger survey

The prevalence of malaria in passengers arriving on Bioko from the mainland was significantly higher than in those leaving Bioko both in adults (35.7 *vs* 22.6%, *p* <0.001) and children under 15 (70.4 *vs* 38.1%, *p* = 0.017) (Table 4). Eighty-six percent of Bioko residents travelling to the mainland said they intended to stay for more than a week and 22% intended to stay for more than seven weeks. In the opposite direction, 85% of mainland residents said they planned to stay on Bioko for more than a week and 26% said they planned to stay more than seven weeks.

**Table 4:** **Malaria prevalence in passengers travelling from Bioko to the mainland and** ***vice versa***

	**Under 15 year olds**			**Aged 15 years or over**		
Direction of travel	Prevalence of *P. falciparum* infection (N)	95% CI	*p*-value	Prevalence of *P. falciparum* infection (N)	95% CI	*p*-value
**Bioko - mainland**	38.1% (63)	26.1-51.2	0.017	22.6% (226)	17.3-28.6	0.001
**Mainland - Bioko**	70.4% (71)	58.4-80.7	ᅟ	35.7% (283)	30.1-41.6	ᅟ

## Discussion

In mainland EG prevalence of malaria has always been substantially higher than on Bioko, ranging from 70% pre-intervention to 59% in 2011, the last year for which prevalence data are available, in two to 14 year olds [10]. The results of this study show that the importation of malaria parasites to Bioko by travellers from mainland EG, where transmission is substantially higher, is a major impediment to reducing the malaria burden on the island. Large numbers of people on Bioko spend time on the mainland which is a significant risk factor for malaria infection, not just for the travellers (Tables 2 and 4) but also for non-travellers in the communities to which travellers return (Table 3). Bioko therefore has both high *vulnerability* and *receptivity* to imported malaria [5,13]. Imported malaria is often primarily regarded as a concern in low-transmission or elimination settings [5,7,13,20]. However, this study shows that imported parasites can be a major factor in malaria transmission even in a high-transmission setting such as Bioko.

Between 2007 and 2010, the percentage of children aged two to 14 surveyed in the BIMCP annual MIS who were reported travelling to the mainland in the previous month was 0.5% or less. This rose to 1.1% in 2011 and 3.2% in 2012. In 2013 and 2014, 6.2 and 6.1%, respectively, of children aged two to 14 were reported travelling to the mainland in the previous eight weeks. If the increase in travel to the mainland seen in the last two years continues then this could further exacerbate the negative impact that travel has already had on malaria control on Bioko by further increasing the vulnerability and receptivity to infection. Moreover, if future malaria control interventions succeed in further reducing infection to pre-elimination levels, then imported malaria would be expected to take on an even greater relative importance than it does now [13], and could fuel transmission even if the reproductive number, R_0_, has declined below 1 [21]. This has been seen on islands which previously had high malaria burdens, such as the Zanzibar archipelago [5,6] and Mauritius [7] but where travel and migration sustained transmission which had been reduced to low levels.

It is not possible to assess the impact of travel to mainland EG on malaria control on Bioko before 2012 from the MIS data because of the small numbers of children sampled who reported travelling in the previous month. However, malaria transmission on mainland EG has been high and stable throughout the lifetime of the BIMCP and, therefore, it is plausible that before 2012 people arriving from the mainland had higher infection rates than those who did not travel.

Table 4 shows that prevalence of malaria in boat passengers leaving the mainland is higher than that of passengers leaving Bioko. A significant proportion of travellers between Bioko and the mainland travel by aeroplane. Airline passengers could well have different socio-economic characteristics to those who travel by boat and consequently have different malaria risks. It would be informative to conduct a similar study on airline passengers. Another possible factor in air travel is sporozoite-positive mosquitoes being transported by aeroplanes, causing so-called airport malaria [1].

A limitation of this study is that it uses observational data. Although many observed confounding variables were adjusted for in this analysis, there could still be residual confounding; but since it is not possible to randomly allocate people to travelling this will be a limitation of any study of this issue. A further limitation of household surveys is that people who are not in their home at the time cannot take part in the survey. Since travellers by definition spend time away from home, this study is likely to have underestimated the proportion of Bioko residents who travelled to mainland EG.

There are a number of strategies through which the effect of imported malaria could be curtailed in Bioko. These broadly fall into three categories: (i) providing protection against malaria for travellers before travelling to the mainland; (ii) clearing parasites in travellers arriving from the mainland; and, (iii) adopting comprehensive malaria control measure in all parts of EG, including the mainland.

The easiest and lowest cost approach to protect prospective travellers to the mainland would be to introduce an information and education campaign about the risks of infection and the need to take precautions. Advice could include using topical repellents, sleeping under bed nets, and taking anti-malarial prophylaxis. Anti-malarial prophylaxis could be sold at socially marketed prices at the port and airport. Since members of wealthier households travel more (Table 1), the financial burden would fall predominantly on those who are better able to pay. However, since malaria importation is a serious public health problem, putting non-travellers at increased risk of malaria, there is a strong case for making anti-malarial prophylaxis available to prospective travellers at no cost.

Clearing parasites from incoming travellers is a complex undertaking facing many pre-elimination countries. A costly approach would entail screening and treating or presumptively treating every person arriving on Bioko from the mainland by boat or plane. The cost of this could be added to the price of the ticket. The effectiveness and acceptability of such approaches would need to be evaluated. Mobile phone data has been used to model the impact of human travel on malaria transmission [5,22]. If these data are available to the BIMCP they could be used to model the potential impact of approaches to reducing importation.

It is well known that malaria transmission is not homogenous and that efficiency of malaria control can be increased by targeting *hotspots*: geographical locations that maintain malaria transmission at higher rates than their surroundings, and *hotpops*: demographic groups that maintain malaria transmission at higher rates than the surrounding population [23,24]. Further research may be able to identify groups of travellers who may be regarded as hotpops to be targeted for parasite clearance. For example, military personnel and their families regularly rotate between Bioko and the mainland and could be a group amenable to presumptive treatment. Similarly, school children are often sent to the mainland for the school holidays.

Ultimately the most effective and sustainable way of preventing the importation of malaria parasites from the mainland would be to reduce the malaria burden on the mainland. This underscores the importance of regional approaches to malaria control as a long-term goal and a necessary strategy for making elimination of malaria sustainable [25].

## Conclusions

This study shows that Bioko residents who travel to mainland EG have a much higher prevalence of malaria than those who do not travel. Moreover, non-travellers are at higher risk of malaria infection if they live in an area with a large number of travellers. The increasing number of travellers from mainland EG are likely to be a growing obstacle to reducing the malaria burden below current levels. Providing information, education and prophylaxis for travellers to the mainland, and clearing parasites in passengers arriving from the mainland should be considered as targeted control measures, supplementary to the BIMCP’s existing interventions. Malaria elimination on Bioko is unlikely to succeed without addressing the high malaria burden on mainland EG.
